# Analytic and Clinical Validation of a Pan-Cancer NGS Liquid Biopsy Test for the Detection of Copy Number Amplifications, Fusions and Exon Skipping Variants

**DOI:** 10.3390/diagnostics12030729

**Published:** 2022-03-17

**Authors:** Audrey Audetat, Chérie Tschida, Sarah Kreston, Adam Stephen, Brittany D’Alessio, Madeline Bondy, Leisa Jackson, Hestia Mellert, Niki Givens, Ubaradka G. Sathyanarayana, Gary A. Pestano

**Affiliations:** Biodesix Inc., 2970 Wilderness Place Suite 100, Boulder, CO 80301, USA; audrey.audetat@biodesix.com (A.A.); cherie.tschida@biodesix.com (C.T.); sarah.kreston@biodesix.com (S.K.); adam.stephen@biodesix.com (A.S.); brittany.dalessio@biodesix.com (B.D.); madeline.bondy@biodesix.com (M.B.); leisa.jackson@biodesix.com (L.J.); hestia.mellert@biodesix.com (H.M.); niki.givens@biodesix.com (N.G.); ubaradka.sathyanarayana@biodesix.com (U.G.S.)

**Keywords:** non-small cell lung cancer (NSCLC), liquid biopsy, cell-free nucleic acid (cfNA), next generation sequencing (NGS), CNA (copy number amplifications), fusions, exon-skipping

## Abstract

Liquid biopsies are an integral part of the diagnosis of cancer. Here, we have extended previous validation studies of a new targeted NGS panel to include the detection of copy number amplifications (CNAs), fusions, and exon skipping variants. Detection of these gene classes included specimens from clinical and healthy donors and cell lines (fusions: ROS1, EML4-ALK, NTRK1; exon skipping: MET exon 14; CNAs: HER2, CDK6, EGFR, MYC, and MET). The limit of detection (LOD) for fusion/skipping was 42 copies (QC threshold was three copies) and was verified using three additional fusion/skipping variants. LOD for CNAs was 1.40-fold-change (QC threshold = 1.15-fold change) and was verified with three additional CNAs. In repeatability and intermediate precision (within lab) studies, all fusion/skipping variants were detected in all runs and all days of testing (n = 18/18; 100%); average CV for repeatability was 20.5% (range 8.7–34.8%), and for intermediate precision it was 20.8% (range 15.7–30.5%). For CNAs, 28/29 (96.6%) copy gains were detected. For CNAs, the average CV was 1.85% (range 0% to 5.49%) for repeatability and 6.59% (range 1.65% to 9.22%) for intermediate precision. The test panel meets the criteria for being highly sensitive and specific and extends its utility for the serial detection of clinically relevant variants in cancer.

## 1. Introduction

Lung cancer accounts for approximately 27% of all cancer-related deaths worldwide [1]. The goal of cancer genetics is to discover genetic aberrations that may predispose cells to undergo oncogenesis. DNA copy number variations (CNV), including amplifications and losses, and gene fusions are important components of genetic variation and can lead to cancer. The continued development of molecular testing using a combination of various tests to reliably screen for the presence of all types of clinically relevant genomic aberrations is critical to advance the field.

A frequent problem that is especially observed in advanced-stage NSCLC patients is the small tissue biopsy size, which can lead to low tumor content. A tissue biopsy sample may also not be representative of the total load and spectrum of mutated cells. However, surgical complications and economic considerations make multiple or serial tissue biopsies impractical in most cases [1,2,3,4]. Additionally, since serial tissue biopsies may increase patient risk and are not economically feasible, diagnostic information at initial diagnosis versus at targeted treatment initiation may be different based on the evolution of the cancer and the use of various treatment modalities, including conventional chemo- or radiotherapy. Thus, several limitations exist with the use of tissue biopsies that may hamper the implementation of various molecular testing modalities. 

Testing of circulating cell-free nucleic acids (cfNA) from the liquid biopsy is a potential solution to the challenges created by the current requirement of tissue biopsies for the diagnosis of cancer, and both regulatory agencies and physician-led associations have provided guidance for the use of liquid biopsies in clinical practice [5,6]. The guidance was initially limited to recommending liquid biopsy diagnosis only when tissue was not available, but liquid biopsy is now considered a complementary method to tissue. If the plasma is negative, a tissue biopsy is recommended when possible [7,8]. This approach leverages the benefits of liquid biopsy while still minimizing the risk of missing actionable variants in those individuals not shedding tumor-derived DNA into their bloodstreams. Current testing options for actionable mutations in NSCLC using cfDNA and a similar ddPCR-based (droplet digital) workflow for rapid result generation include the epidermal growth factor receptor (EGFR) sensitizing mutations, including ΔE746-A750, L858R, T790M, the KRAS proto-oncogene variants KRAS G12C/D/V, and B-Raf proto-oncogene variant BRAF V600E [4]. Although not as widely adopted by the field currently, circulating tumor-derived messenger RNA (mRNA) isolated from plasma can also provide important clinical information, and the detection of RNA fusions including ALK, ROS1, and RET has been demonstrated and is being utilized [1,4,9].

Gene fusions have emerged as an important class of markers for precision medicine in solid tumors. Fusion transcripts or aberrantly spliced transcripts are an important class of oncogenic somatic alterations. Among targetable alterations with approved drugs in ADC (advanced lung adenocarcinoma), there are different fusion proteins leading to constitutively activated kinases. Patients with ADC harboring anaplastic lymphoma kinase (ALK), ROS proto-oncogene 1 (ROS1), rearranged during transfection proto-oncogene gene (RET), and neurotrophin kinase (NTRK) gene rearrangements can benefit from a treatment with tyrosine kinase inhibitors (TKIs); therefore, sensitive, specific, accurate, and robust molecular testing is essential [10]. Transforming rearrangements of the anaplastic lymphoma kinase (ALK) gene are present in 3–6% of lung adenocarcinomas (LUADs), and these tumors are responsive to crizotinib. Rearrangements of ROS1 and RET have also been found in LUADs at a prevalence of 1–3% and are responsive to crizotinib and multi-kinase inhibitors cabozantinib and vandetanib, respectively. Targeted RNA-Seq is an emerging form of testing for gene fusions with distinct advantages over IHC and FISH, including better sensitivity, specificity, and multiplexing density [11].

Next-generation sequencing (NGS) technology has contributed to a paradigm shift in molecular pathology from single-gene tests to multi-gene panels [11,12]. These panels have been widely used in basic research and in clinical diagnostics. Clinical sequencing of tumor DNA in tissue has received the greatest attention, with an emphasis on the detection of hotspot single nucleotide variants (SNVs), small insertions and deletions (INDELs), and copy number variants (CNVs; including amplification and losses) that confer sensitivity to targeted therapies. For example, somatic variations in exons 18–21 of EGFR occur in approximately 10–15% of non–small cell lung cancer (NSCLC) tumors and are sensitizing to first-generation tyrosine kinase inhibitors (TKIs) erlotinib and gefitinib [10]. Routine profiling of tumor DNA variation for established and emerging drug targets is now possible in clinical reference labs through validated NGS panels based on hybridization capture or targeted amplicon sequencing.

In contrast to NGS assays developed for SNVs, INDELs, and CNVs, targeted NGS assays developed for gene fusion detection are predominately based on RNA-Seq. RNA-based testing can be more sensitive, efficient, and functionally definitive considering that many DNA variants (e.g., multiple intronic breakpoints) give rise to the same oncogenic transcript. In addition, a targeted RNA-Seq is capable of detecting additional classes of clinically relevant RNA variations, including aberrant splice variants such as the exon 14 skipped isoform of MET, which leads to a constitutively activated form of cMET that confers sensitivity to crizotinib.

We have previously reported on a highly sensitive and robust blood-based ddPCR (droplet digital PCR) assay to identify EGFR-sensitizing mutations (L858R and E746_A750), the EGFR-resistance mutation (T790M), KRAS G12C/D/V, BRAF V600E in circulating DNA, and EML4-ALK variants 1/2/3 in circulating RNA [4,9]. We subsequently extended these methods to include the detection of higher-order multiplexed RNA targets for ROS1 and RET, covering eight fusion variants within each assay [9]. These advances led to the development of a rapid, sensitive, specific, and reproducible technique for the detection of these fusion variants from the plasma of patients previously diagnosed with NSCLC [9]. Further, we have reported and deployed these and multiple other targeted liquid biopsy-based test systems [4,9,12,13,14] for use in clinical testing. The current focus of this report is on a new targeted NGS panel that can detect alterations in 52 genes, and it includes the identification of substitutions, including single nucleotide variants (SNV), multiple nucleotide variants (MNV), insertions, deletions, copy number amplifications (CNAs), and gene fusions/skipping [12].

This manuscript focuses on the extension and validation of performance verification for the detection of CNAs and fusion/skipping variant types within the GeneStrat NGS™ Genomic Test [12]. Specifically, apart from SNV and INDELSINDEL, the panel is also designed to detect 12 genes with potential for CNA and 13 genes with fusion/skipping variants [12 fusion genes (96 specific fusion variants total) and MET exon 14 skipping]. All targets are of relevance in oncology. We describe the performance of our batch process QC metrics, as well as analytic sensitivity and specificity, and within lab intermediate precision and repeatability validation studies. Studies were performed using a combination of donor specimens and contrived specimens generated by admixing retrospectively-banked clinical specimens with tumor-derived immortalized cell-lines.

## 2. Materials and Methods

### 2.1. Specimens Collection and Preparation

Remnant clinical specimen use in these studies is considered exempt research under 45 CFR 46.104(d)(4), which is the relevant exemption section to the Common Rule (45 CFR Section 46). Normal healthy donor specimens were collected under IRB-approved protocol (BDSX-CD-007; ADVARRA). Whole blood was collected into a Cell-Free DNA Blood Collection Tube^®^ (Streck, La Vista, NE, USA) and held at ambient temperature for no longer than seven days [4,9,12,13,14,15,16,17]. Whole blood was spun at 1900× *g* for 10 min, and then plasma was separated and super-spun at 16,000× *g* for 10 min [4,9,12]. Extraction was performed using the QIAamp Circulating Nucleic Acid Kit (Qiagen, Hilden, Germany), using up to 10 mL of plasma, and it was eluted in a final volume of 100 μL. Following extraction, total nucleic acid (TNA) was concentrated using the RNA Clean-up and Concentration Micro Elute kit (Norgen, Thorold, Ontario, Canada), eluted in 26 μL and quantified using the Qubit dsDNA High-Sensitivity Assay Kit (Thermo Fisher Scientific, Waltham, MA, USA).

Contrived specimens were generated by extracting intracellular DNA or RNA (Qiagen, Hilden, Germany) from tumor-derived cell lines containing desired variant types and spiked into a background of cell-free nucleic acid. Cell lines were selected based on their relevant variant content. HCC78 harboring SLC34A2-ROS1 (Cat #: CSC-C0569; Creative Bioarray, NY, USA), CRL-5935 harboring EML4-ALK (Cat #: NCI-H2228; ATCC, Manassas, VA, USA), KM12 (acquired from MD Anderson Cancer Center, Houston, TX, USA) harboring TPM3-NTRK1, and HTB-178 (Cat #: NCI-H596, ATCC) harboring MET exon 14 skipping were used for fusions and exon skipping. CRL-5928 (Cat #: NCI-H2170, ATCC) containing ERBB2/HER2 gain, SK-BR-3 (Cat #: HTB-30, ATCC) containing ERBB2/HER2, CDK6, EGFR and MYC gains, HCC-827 harboring EGFR gain (Cat #: CRL-2868DQ, ATCC), and CRL-5909 harboring MET gain (Cat #: NCI-H1993, ATCC) were used for CNAs. Intracellular DNA and RNA was eluted in 200 µL AE Buffer and 50 µL nuclease-free water, respectively, and quantified using Qubit dsDNA High-Sensitivity Assay and Qubit RNA High-Sensitivity Assay Kit (Thermo Fisher Scientific). An amount of 20 ng of intracellular DNA and 5 ng of intracellular RNA was input into reverse transcription reactions. To generate contrived specimens, each cell-line extract was admixed into remnant, de-identified pooled cfNA background specimens obtained from patients previously diagnosed with NSCLC. Cell-line cDNA was admixed at varying volumes of cfNA to generate high, medium, and low concentration-contrived specimens before use in library preparation for NGS.

### 2.2. Next Generation Sequencing

Reverse transcription, library preparation, quantification, pooling, templating, sequencing, and bioinformatic analysis was performed as previously described [12]. Briefly, 20 ng of nucleic acids were used as input to the reverse transcription reaction and followed by library preparation using the Oncomine Pan-Cancer Cell-Free Assay (Thermo Fisher Scientific (TFS), San Francisco, CA, USA) [18] according to the manufacturer’s instructions. The target regions in specimens were amplified with barcode-adapted primers, purified, and size selected with AMPure XP Reagents (Beckman Coulter). Library quantification was performed using the Ion Library TaqMan Quantitation Kit (Thermo Fisher Scientific) on the Light Cycler 96 (Roche Diagnostics, Indianapolis, IN, USA). Libraries were diluted to a final concentration of 50 pM, which is the minimum required based on established QA criteria, and up to eight libraries were pooled at the equimolar concentration for chip templating. Ion 550™ Chips were templated on the Ion Chef™ instrument (Thermo Fisher Scientific) and sequenced on the Ion GeneStudio™ S5 Prime (Thermo Fisher Scientific) using the Ion 550 Kit-Chef as described by the manufacturer (Thermo Fisher Scientific). Sequencing data were analyzed using predefined metrics in Torrent Suite Software version 5.12 and Ion Reporter™ version 5.10 (Thermo Fisher Scientific). Variant calling was performed using the Oncomine™ TagSeq Pan-Cancer Liquid Biopsy w2.1—Single Sample workflow within the Ion Reporter (Thermo Fisher Scientific).

### 2.3. Quality Control (QC) Metrics

Sample level QC thresholds were applied as follows: ≥10 million total mapped reads, ≥95% coverage uniformity, ≥1500 median molecular coverage, ≥80% molecular-based uniformity, ≥80 bp mean read length, and ≥80% of bases with AQ20 (read error is 1% or less). Specimens that did not meet these criteria were not included in this analysis. Additional metrics were applied to verify adequate panel performance for the detection of CNA and fusion/skipping variant types as below. For CNA, the manufacturer (Thermo Fisher Scientific) set the sample-level QC criteria of the median absolute pairwise difference (MAPD) to <0.4. In addition, the CNA variant region must meet a *p*-value < 10^−5^ and a CNA ratio >1.15 to pass the bioinformatic QC threshold. The CNA ratio call thresholds were previously derived empirically by the manufacturer using plasma samples from healthy donors with normal CNA status [19]. To pass the bioinformatic QC threshold for CNAs, a specimen must therefore contain ≥1.5-fold gain in that variant. We note that the Oncomine™ Pan-Cancer Cell-Free Panel does not report copy number loss.

For fusions/skipping variants, the Oncomine™ Pan-Cancer Cell-Free Panel includes two target genes, TBP and HMBS, and two MET wild-type exon junction targets. At least one control (TBP or HMBS) and at least one wild-type MET exon junction (E6-E7 or E11-E12) must have a molecular count of ≥3 molecular families to pass fusion/skipping QC criteria. To pass the bioinformatic QC threshold for fusions or skipping, a specimen must then contain ≥3 molecular families/molecular copies of that fusion or skipping variant.

### 2.4. Batch Process and Controls

An analytic positive control for the test was generated and used to monitor each batch for quality assurance. The positive control was a mixture of total genomic DNA extracted from tumor-derived cell-line harboring copy number variants and synthesized gene segments (gBlocks; Integrated DNA Technologies; Coralville, IA, USA), representing EGFR L858R, EGFR T790M, EGFR ∆E746-A750, KRAS G12C, and BRAF V600E, in a background of sonicated/fragmented human genomic DNA designed to mimic cfDNA prepared as described previously [4,12]. The addition of in vitro transcribed RNA generated from gene segments with T7 promoter sequences (gBlocks; Integrated DNA Technologies) representing EML4-ALK, CCDC6-RET, and CD74-ROS1 and total RNA extracted from a tumor-derived cell-line provided the necessary process controls for monitoring of the fusion/skipping variant types. This mixture was added to pooled plasma and processed alongside each batch of clinical specimens, beginning at the extraction step, and brought through the entirety of the workflow. Only those batches that tested positive for all variant types in the positive control were considered passing. Additionally, a no template control (NTC) was run with each batch of clinical samples. The established quality control criteria for the NTC were ≤6 million total mapped reads or ≤45 bp mean read length as well as being void of detected variants.

## 3. Results

### 3.1. Process Control Verification

To maintain control of the NGS test workflow, the positive control was designed to represent both the previously validated SNV and INDELS (data not shown), as well as the new variant types of interest. Preparation of the positive control included the use of components including synthetic fragments, tumor-derived cell-line material, and normal pooled plasma, as described above in the methods section.

**Figure 1:**
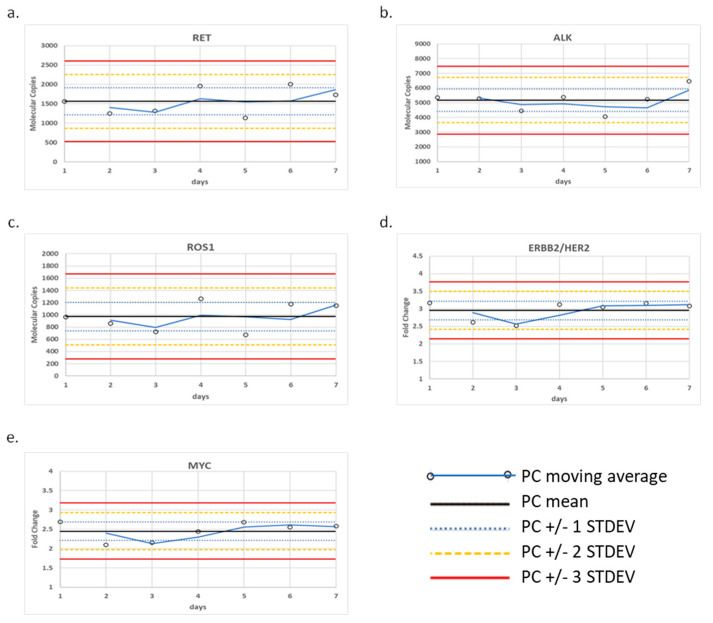
Control charts for CNA and fusion/skipping variant types. (**a**) CCDC6-RET fusion, (**b**) EML4-ALK fusion, (**c**) CD74-ROS1 fusion, (**d**) ERBB2/HER2 amplification, and (**e**) MYC amplification detected within the positive control over seven days of testing.

The positive control was tested over seven days and the fusion variants and CNAs were monitored using control charts (Figure 1). To monitor fusion transcripts, we generated molecular reads that represent the fusion transcript of interest relative to an average of the four internal process controls designed within the panel (TBP, HMBS, MET E6:E7, and MET E11:E12). Using this calculation, we established QC limits for each of three control transcripts based on a ± 3 standard deviation (St. Dev.) from the mean, which were determined to be the following ranges: 525–2605 reads for CCDC6-RET, 2880–7474 reads for EML4-ALK, and 276–1671 reads for CD74-ROS1. Similarly, we established QC limits for CNA based on a ± 3 St. Dev. from the mean of their fold-change, which were determined to be the following ranges: 2.14–3.77-fold change for ERBB2/HER2 and 1.73–3.18-fold change for MYC.

### 3.2. Analytic Sensitivity

Analytic sensitivity for fusions/exon skipping and CNAs was established using a five-point dilution series of HCC78 cell-line (Cat #: CSC-C0569; Creative Bioarray, NY, USA) harboring the SLC34A2-ROS1 fusion variant and the CRL-5928 cell-line (Cat #: NCI-H2170, ATCC) containing ERBB2/HER2 CNA, respectively. Each cell line was admixed into pooled cfNA background from patients previously diagnosed with NSCLC. Fusion/skipping initial analytic sensitivity yielded a 100% detection rate of the expected variant at all dilutions (Table 1). To evaluate the accuracy of the test for the detection of fusions, average molecular coverage at the lowest dilution (dilution 5) was determined as 42 copies, which aligns with the 34 copy LOD previously established by the manufacturer [19]. All assays passed the pre-set QC thresholds (greater than three copies detected for fusions and 1.15-fold gain for CNA) also required in the Oncomine Pan-Cancer assay bioinformatic pipeline.

CNA analytic sensitivity was determined consistently at dilutions 1 through 3 (100% detection of expected variants) but was not detected in dilution 4 or 5 (Table 2). To evaluate the accuracy of the test for the detection of CNA, average fold-change at the lowest dilution (3) was determined as 1.40 (cv = 1.4%), which is aligned with the 1.34-fold LOD previously established by the manufacturer and passes the bioinformatic QC threshold of 1.15-fold gain [19].

To verify the analytic sensitivity using additional variants, six additional cell lines were diluted to the established LOD in cfNA matrix background and evaluated for the ability to pass the relevant QC threshold qualification at or near the target LOD. Three cell-lines targeted the LOD determined above: average 1.4-fold (cv = 1.4%) change for CNA and an additional three targeted an average of 42 copies (cv = 23%) of molecular coverage for fusion/skipping. The respective bioinformatic QC thresholds required for the detection of CNA and fusions are ≥1.15 and ≥3 copies, respectively.

**Figure 2:**
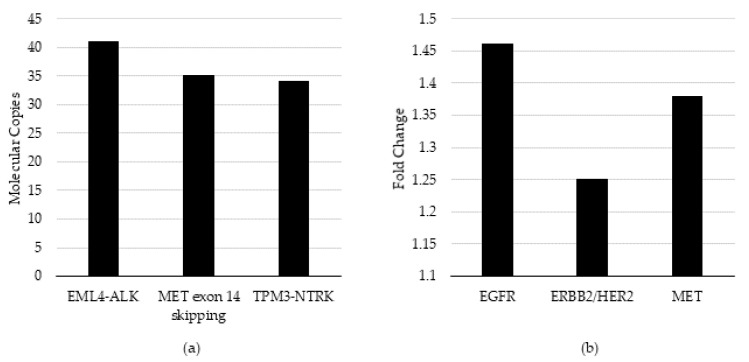
QC threshold evaluation and limit of detection verification. (**a**) Fusion/skipping variant types measured as molecular copies and (**b**) CNA variant types measured as fold-change were evaluated using the pre-set QC thresholds in the Oncomine Pan-Cancer assay bioinformatic pipeline and assessed detection at or near the pre-determined LOD.

Specifically, the HCC-827 cell-line DNA (Cat #: CRL-2868DQ, ATCC) containing EGFR amplification, SK-BR-3 (Cat #: HTB-30, ATCC) containing ERBB2/HER2 amplification, and CRL-5909 (Cat #: NCI-H1993, ATCC) containing MET amplification were evaluated for CNA. The CRL-5935 cell-line (Cat #: NCI-H2228; ATCC, VA) containing EML4-ALK fusion, HTB-178 (Cat #: NCI-H596, ATCC) containing MET exon 14 skipping, and KM12 (acquired from MD Anderson Cancer Center (Houston, TX) under a Material Transfer Agreement) containing TPM3-NTRK1 fusion were evaluated for fusions. 100% of expected variants were detected above the analytic QC thresholds in the bioinformatic pipeline and for detection at or near the assay LOD (Figure 2). We noted, from the control charts generated over seven days of testing, that the variance around ERBB2/HER2 and MET varied from day to day, although within +/− 3 S.D. 2.14–3.77-fold change for ERBB2/HER2 and 1.73–3.18-fold change for MYC (Figure 1).

### 3.3. Analytic Specificity

For each variant type, specificity was calculated for each individual specimen and for all specimens combined (Table 3). Total targets that were assayed by the GeneStrat NGS Genomic Test for each variant type were based on the fusion/skipping content (95 fusion/skipping targets) and CNA genes candidates (12 CNAs) within the panel design and bioinformatic workflow provided by Thermo Fisher Scientific. The NHD (Normal Healthy Donor) specimens lacked detectable variants in each variant type with no exceptions. These data demonstrate that the reference range was appropriately below the assay cutoff. The specificity for each variant type was 100% for fusion/skipping (greater than 42 copies) and 100% for CNAs (greater than a 1.4-fold change). Although not the focus of this manuscript, we noted 100% specificity of the 979 SNV and INDEL targets assayed in the panel across all 12 donors as well (data not shown). Overall, the specificity within the study of these twelve specimens when evaluating all variant types (SNV, INDEL, CNA, fusions, and exon-skipping) was 100%. In conclusion, the GeneStrat NGS Genomic Test is highly specific and suitable for the intended use in patients previously diagnosed with NSCLC.

### 3.4. Repeatability and Intermediate Precision

Repeatability (within runs) and intermediate precision (within-lab; between runs) was evaluated using a high, medium, and low frequency positive specimen for fusion/skipping and two high and three low frequency positive genes represented by two specimens for CNAs for a total of five specimens. The repeatability study evaluated each of the five specimens in triplicate within the same run. Two CNAs were evaluated on day 1 and three fusion/skipping specimens on day 2. The intermediate precision study evaluated each of the specimens across four runs, each on a different day. Specifically, two different operators, two different instruments, and unique barcodes were used. CNA was evaluated using days 1, 3, 4, and 5; fusion/skipping was evaluated using days 2, 3, 4, and 5.

For each variant type, CNA, and fusion/skipping, positive specimens were evaluated. Three specimens at high, medium, and low concentrations were evaluated, each with a single fusion. Two specimens containing five CNAs (two high and three low) were evaluated. Intermediate precision testing was performed over five total days, four for fusion/skipping and an overlapping four for CNAs.

**Figure 3:**
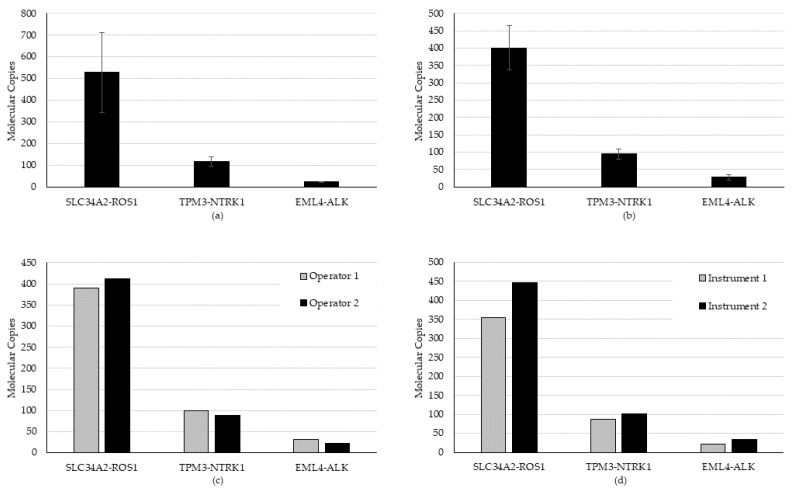
Repeatability and intermediate precision study results for the fusion/skipping variant type. (**a**) Repeatability, (**b**) inter-day, (**c**) inter-operator, and (**d**) inter-instrument variability were evaluated using a high, medium, and low contrived specimen.

All expected fusion/skipping variants were detected in all runs and all days of testing (n = 18/18), generating a hit rate of 100%. An average CV of 20.5% (range 8.7% to 34.8%) for repeatability and 20.8% (range 15.7% to 30.5%) for intermediate precision was established for fusion/skipping (Figure 3).

**Figure 4:**
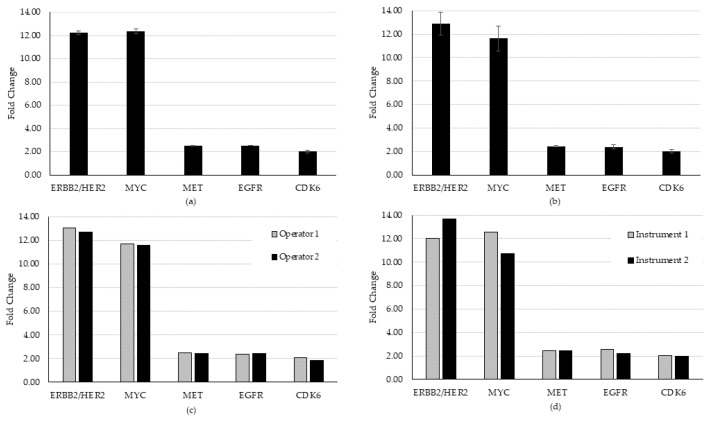
Repeatability and intermediate precision study results for the CNA variant type. (**a**) Repeatability, (**b**) inter-day, (**c**) inter-operator, and (**d**) inter-instrument variability were evaluated using two contrived specimens representing five unique amplification events. Overall, the study demonstrated acceptable consistency in the detection of both the CNA and fusion/exon-skipping variant types in biologically relevant specimens.

For CNA evaluation in precision, all CNAs were detected in all specimens, except for one that continually failed QC and was removed from further analysis because of pre-analytic concerns in the preparation of that specimen. Of the remaining samples, 28 of 29 CNAs were detected, generating a hit rate of 96.6%. On the one miss, we observed that an expected CDK6 gain did not meet the bioinformatic QC threshold of 1.15-fold. This is likely due to sensitivity to template preparation for low frequency calls for this variant. All other CNAs present in that specimen (higher frequency) were detected above the cutoff. An average CV of 1.85% (range 0% to 5.49%) for repeatability and 6.59% (range 1.65% to 9.22%) for intermediate precision was established for the detected CNAs (Figure 4).

## 4. Discussion

Liquid biopsy with circulating free nucleic acid (circulating DNA and RNA) profiling by next-generation sequencing holds great promise for precision medicine in the therapeutic management of cancer patients. It relies on the basis that circulating nucleic acids derived from tumor cells represent the real-time status of the tumor genome, which contains information that may relate to genetic alterations. Liquid biopsy complements traditional tissue biopsies in several ways: it is a less demanding procedure for patients, is minimally invasive, accommodates frequent sampling, and has inherently less sampling bias as compared to tissue biopsies. The profiling cfNA with NGS for cancer diagnosis can potentially help to detect cancer earlier, effectively identify actionable mutations, as well as help with the prognosis of cancer patient outcomes [20,21,22].

Liquid biopsy provides physicians with a complementary approach to tissue biopsies that benefits from a minimally invasive blood draw, amenability to serial testing, and rapid turnaround time [2,4,9,12]. We have previously validated SNV and INDEL variant types and published the initial validation of the GeneStrat NGS genomic test panel [12]. Here, our goal was to further verify the performance of the cell-free circulating nucleic acid NGS panel for use in a regulated clinical laboratory for the routine detection of CNA and fusion/skipping variant types. We initially established the processes for the controls that would be used to monitor batch performance for the clinical assay (Figure 1). By admixing representative templates for each variant type using cell lines and synthetic gene fragments, we were able to build a clinically relevant workflow process that is suited to the regulatory requirements that govern testing in our centralized laboratory.

Three studies were conducted to support the assay validation, which included analytic sensitivity, specificity, and precision. Accuracy for the detection of CNAs, fusions, and exon-skipping was determined during analytic sensitivity evaluation. It related the respective QC thresholds (bioinformatic pipeline) and actual LOD to the validation studies previously conducted by the manufacturer [19] and from literature reviews of studies of the cell line specimens conducted by external investigators (Supplementary Table S1 and references therein, [1,23,24,25,26]). Our results were consistent with those previously determined for the detection of the gene classes investigated in our study (Supplementary Table S1).

In summary, the limit of detection, repeatability, and intermediate precision acceptance criteria were established and verified for two additional variant types not previously evaluated in our laboratory [12,14], i.e., CNA and fusion/skipping. The LOD for fusion/exon-skipping was established at a molecular coverage of 42 copies, using a serial dilution of a single fusion variant, and it was verified by the detection of three additional fusion/exon-skipping variants at or near the LOD. The LOD for CNA was established at 1.40-fold-change using a serial dilution of a single CNA, and it was verified with the detection of three additional CNAs at or near the LOD (Figure 2). All test verifications passed the pre-determined QC thresholds of 1.15-fold gain and three copies for the detection of CNAs and fusions, respectively [19]. All assays were within the day-to-day variance limits of the assays established using controls charts (Figure 1).

Precision testing was performed over five total days, four for fusion/skipping (Figure 3) and an overlapping four for CNAs (Figure 4). All expected fusion/skipping variants were detected above the bioinformatic QC threshold as required for all runs and all days of testing (n = 18/18) generating a hit rate of 100%. An average CV of 20.5% (range 8.7% to 34.8%) for repeatability and 20.8% (range 15.7% to 30.5%) for intermediate precision was established for fusion/skipping. One CNA (CDK6) specimen failed to meet the required QC threshold of a 1.15-fold gain upon both the first run and a rerun and was thus censored from the precision analysis for that day. Of the remaining samples, 28 of 29 CNVs were detected, generating a hit rate of 96.6%. An average CV of 1.85% (range 0% to 5.49%) for repeatability and 6.59% (range 1.65% to 9.22%) for intermediate precision was established for detected CNAs.

The specificity of the test was evaluated using consented healthy donors with no previous diagnosis of cancer (HOPE Study Protocol, BDSX-CD-007). All donor samples passed the internal fusion/skipping and CNA control evaluations, and none were detected as harboring fusion/skipping variants or CNA above the LOD. These results support the highly sensitive and specific detection of the additional variant classes in the GeneStrat NGS Genomic test and complement previous results showing the same for the detection of SNVs and INDELS [4,12,19].

Analysis of circulating tumor nucleic acids in the plasma of NSCLC patients is the most documented form of liquid biopsy and provides a molecular profile of the tumor without an invasive tissue biopsy. In a study conducted by Papadopoulou et al. [27], liquid biopsy analysis was requested by the referring physicians in 121 NSCLC patients at diagnosis and was performed using a sensitive NGS assay. At least one mutation was identified in almost 49% of the cases by the NGS approach in NSCLC patients analyzed at diagnosis. In 36 cases with paired tissue available, a high concordance of 86.11% was observed for clinically relevant mutations, with a positive predictive value (PPV) of 88.89%. Furthermore, a concordance rate of 82% between cobas^®^ real time PCR and the NGS approach for the *EGFR* sensitizing mutations in exons 18, 19, and 21 was observed in patients with acquired resistance to EGFR TKIs, while this concordance was 94% for the p.T790M mutation, with NGS being able to detect this mutation in three additional patients [27]. This study, in agreement with the current study, reinforces the feasibility of circulating tumor nucleic acids (ctNA) analysis as a tumor biopsy surrogate in clinical practice for NSCLC personalized treatment decision making.

The detection of gene fusion events is important for the detection and management of malignant cancer. In addition to ALK, RET, NTRK1, and ROS1, fusions involving FGFR1/2/3 and NRG1 genes have been reported in NSCLC among other cancers and represent emerging therapeutic targets. A related study conducted in pediatric solid tumors [28] has described the validation of a next-generation sequencing assay for the multiplex detection of gene fusions. Here, the authors reported on 24 previously characterized specimens. Twenty specimens had one or more previously described fusion events, and four specimens were negative for any fusion events. The accuracy across specimens was 100% (20 of 20 specimens). The analytical sensitivity and specificity were both recorded at 100%. Inter-day reproducibility for fusion events was 94%; in comparison, intra-day reproducibility was 90%. This multiple-gene fusion assay demonstrated appropriate sensitivity, specificity, and accuracy for clinical use. Even though this study was conducted in solid tumors, it is consistent with the data generated here, further emphasizing the feasibility of the use of blood-based tests as a surrogate for tumor tissue biopsies in clinical practice.

Our earlier publication [4], where test development using ddPCR included method and clinical validation using samples from donors with (n = 219) or without (n = 30) cancer, showed that clinical sensitivity and specificity for each variant ranged from 78.6% to 100% and 94.2% to 100%, respectively. The performance of the targeted approach for liquid biopsies was further evaluated in another of our previous studies [12] using this amplicon-based NGS panel to validate somatic nucleotide variants (SNVs) and INDELS using contrived and retrospectively-collected clinical specimens. The detection of SNV and INDELS was 97.7–100%, concordant with orthogonal droplet digital PCR (ddPCR) tests [12]. Together, these studies further support the use of targeted molecular testing using liquid biopsies in the clinical management of cancer patients.

## 5. Conclusions

Determination of the genetic identity of circulating tumor DNA targets in liquid biopsy using NGS technology and ddPCR-based genotyping assays is suitable for implementation in routine clinical decision making for advanced NSCLC patients. We have extended our validation studies with previously conducted ddPCR [4,9,13,14] and NGS [12] to additional genes and variants, including CNA and fusion/skipping, using the latter pan-cancer NGS assay. The circulating tumor nucleic acids in the plasma of NSCLC patients are commonly used for a form of liquid biopsy. Serial analysis of the molecular profile of the tumor DNA in plasma without need for repeated invasive tissue biopsy holds promise for better personalized treatment decision making for patients afflicted with cancer. This study indicates the feasibility of circulating free nucleic acid analysis as a tumor biopsy surrogate in clinical practice. Coupled with NGS techniques that can reliably detect tumor-derived genetic aberrations and structural variants, liquid biopsies have the potential to provide clinically relevant information both before and after targeted treatment of patients with cancer. Further studies will continue to address the complexities inherent in the broader patient population not addressed in this study, including test performance in various stages of cancer (early and late), as well as the impact of comorbidities. Our studies have highlighted approaches to test, verify, and validate the suitability of this panel for clinical testing for the rapid and serial detection of SNV, INDELS, fusions/exon-skipping, and CNA in a certified clinical laboratory.

## Figures and Tables

**Table 1:** Analytic sensitivity for the fusion/skipping variant type.

Dilution	Replicate	Detected/Not Detected above Bioinformatic Analysis Cutoff (≥3 Copies)	SLC34A2-ROS1 Molecular Coverage	Average	CV
D1	R1	Detected	549		
	R2	Detected	652	601	12.1%
D2	R1	Detected	272		
	R2	Detected	321		
	R3	Detected	287	293	8.6%
D3	R1	Detected	158		
	R2	Detected	171		
	R3	Detected	141	157	9.6%
D4	R1	Detected	66		
	R2	Detected	98		
	R3	Detected	81	82	19.6%
D5	R1	Detected	49		
	R2	Detected	46		
	R3	Detected	31	42	23.0%

**Table 2:** Analytic sensitivity for the CNA variant type.

Dilution	Replicate	Detected/Not Detected above Bioinformatic Analysis Cutoff (≥1.15-Fold Gain)	ERBB2/HER2 Fold Change	Average	CV
D1	R1	Detected	5.64		
	R2	Detected	5.08	5.36	7.4%
D2	R1	Detected	2.27		
	R2	Detected	2.35		
	R3	Detected	2.27	2.30	2.0%
D3	R1	Detected	1.4		
	R2	Detected	1.42		
	R3	Detected	1.38	1.40	1.4%
D4	R1	None detected	Not detected		
	R2	None detected	Not detected		
	R3	None detected	Not detected	Not detected	Not detected
D5	R1	None detected	Not detected	Not detected	Not detected
	R2	None detected	Not detected		
	R3	None detected	Not detected		

**Table 3:** Assay specificity in normal healthy donor specimens (NHD).

Sample ID	Fusion/Skipping			CNA		
	Assayed	Detected	Specificity	Assayed	Detected	Specificity
NHD1	95	0	100%	12	0	100%
NHD2	95	0	100%	12	0	100%
NHD3	95	0	100%	12	0	100%
NHD4	95	0	100%	12	0	100%
NHD5	95	0	100%	12	0	100%
NHD6	95	0	100%	12	0	100%
NHD7	95	0	100%	12	0	100%
NHD8	95	0	100%	12	0	100%
NHD9	95	0	100%	12	0	100%
NHD10	95	0	100%	12	0	100%
NHD11	95	0	100%	12	0	100%
NHD12	95	0	100%	12	0	100%
Overall	1140	0	100%	144	0	100%

## Data Availability

The authors confirm that the data supporting the findings of this study are available within the article and its Supplementary Materials.

## Supplementary Materials

The following supporting information can be downloaded at: https://www.mdpi.com/article/10.3390/diagnostics12030729/s1, Table S1: Reference Cell line information.
