# Supplementary material for: Analytic and Clinical Validation of a Pan-Cancer NGS Liquid Biopsy Test for the Detection of Copy Number Amplifications, Fusions and Exon Skipping Variants
Source: Diagnostics (Basel). 2022 Mar 17;12(3):729. doi: 10.3390/diagnostics12030729 (PMC8947661; doi:10.3390/diagnostics12030729)
Supplement: Supplementary file 1 [file diagnostics-12-00729-s001.zip › diagnostics-1588672-supplementary.pdf]

Supplementary Data

Table S1. Reference Cell line information

Cell line designation	GeneStrat NGS Test Result	Comparator Result (NGS or other method)	Cell line citation in literature
Cell line #1 (CRL-5935)	ALK-fusion detected (EML4-ALK)	Detected by NGS All-in-one Transcriptome based Assay [1]	NCI-H2228 [H2228] (ATCC CRL-5935)
Cell line #2 (HTB-178)	MET exon 14 skipping	Detected by NGS All-in-one Transcriptome based Assay [1]	NCI-H596 [H596]-HTB-178™ (ATCC)
Cell line #3 (KM12)	TPM3-NTRK1 fusion	Detected by NGS All-in-one Transcriptome based Assay [1]	KM12 (ATCC)
Cell line #4 (HCC78)	SLC34A2-ROS1 fusion	Detected by Mass Spectrometry Assay [23]	HCC78 (Creative Bio-array)
Cell line #5 (CRL-5928)	ERBB2/HER2 CNV	Detected by NGS and qPCR [24]	NCI-H2170 [H2170] CRL-5928™ (ATCC)
Cell line #6 (HCC-827)	EGFR amplification	Detected by NGS and qPCR [24]	HCC827 CRL-2868™ (ATCC)
Cell line #7 (SK-BR-3)	ERBB2/HER2 amplification	(FISH)-based method to determine HER-2 amplification in breast cancer CTCs [25]	SK-BR-3 [SKBR3] HTB-30™ (ATCC)
Cell line #8 (CRL-5909)	MET amplification	Detected by qPCR [26]	NCI-H1993 [H1993] (ATCC)

Audetat A. etal.
